# An Integrated Analysis of MicroRNA and mRNA Expression Profiles to Identify RNA Expression Signatures in Lambskin Hair Follicles in Hu Sheep

**DOI:** 10.1371/journal.pone.0157463

**Published:** 2016-07-12

**Authors:** Xiaoyang Lv, Wei Sun, Jinfeng Yin, Rong Ni, Rui Su, Qingzeng Wang, Wen Gao, Jianjun Bao, Jiarui Yu, Lihong Wang, Ling Chen

**Affiliations:** 1 College of Animal Science and Technology, Yangzhou University, Yangzhou, China; 2 Animal Science and Veterinary Medicine Bureau of Suzhou City, Suzhou, China; Kunming University of Science and Technology, CHINA

## Abstract

Wave patterns in lambskin hair follicles are an important factor determining the quality of sheep’s wool. Hair follicles in lambskin from Hu sheep, a breed unique to China, have 3 types of waves, designated as large, medium, and small. The quality of wool from small wave follicles is excellent, while the quality of large waves is considered poor. Because no molecular and biological studies on hair follicles of these sheep have been conducted to date, the molecular mechanisms underlying the formation of different wave patterns is currently unknown. The aim of this article was to screen the candidate microRNAs (miRNA) and genes for the development of hair follicles in Hu sheep. Two-day-old Hu lambs were selected from full-sib individuals that showed large, medium, and small waves. Integrated analysis of microRNA and mRNA expression profiles employed high-throughout sequencing technology. Approximately 13, 24, and 18 differentially expressed miRNAs were found between small and large waves, small and medium waves, and medium and large waves, respectively. A total of 54, 190, and 81 differentially expressed genes were found between small and large waves, small and medium waves, and medium and large waves, respectively, by RNA sequencing (RNA-seq) analysis. Differentially expressed genes were classified using gene ontology and pathway analyses. They were found to be mainly involved in cell differentiation, proliferation, apoptosis, growth, immune response, and ion transport, and were associated with MAPK and the Notch signaling pathway. Reverse transcription-polymerase chain reaction (RT-PCR) analyses of differentially-expressed miRNA and genes were consistent with sequencing results. Integrated analysis of miRNA and mRNA expression indicated that, compared to small waves, large waves included 4 downregulated miRNAs that had regulatory effects on 8 upregulated genes and 3 upregulated miRNAs, which in turn influenced 13 downregulated genes. Compared to small waves, medium waves included 13 downregulated miRNAs that had regulatory effects on 64 upregulated genes and 4 upregulated miRNAs, which in turn had regulatory effects on 22 downregulated genes. Compared to medium waves, large waves consisted of 13 upregulated miRNAs that had regulatory effects on 48 downregulated genes. These differentially expressed miRNAs and genes may play a significant role in forming different patterns, and provide evidence for the molecular mechanisms underlying the formation of hair follicles of varying patterns.

## Introduction

Persian lamb skin is one of the “three pillars” of the international fur market. Its trade volume is 11,000,000 to 13,000,000 tons, accounting for 15%–20% of the world’s fur market in 2007. The Karakul breed of sheep is well known throughout the world, particularly for its lambskin that brand name is “Bukhara”, which is mostly black and gray, and represents about 50% of the world’s lambskin production. To increase the variety of colors in lambskin, black lambskin from Karakul sheep is usually decolorized and dyed with other colors, but the process of decolor can significantly affect its quality. The cultivation of sheep with high-quality white lambskin has been performed for centuries.

Hu sheep are a breed with white lambskin that is unique to China, and regarded as a protected breed by the Chinese government. Lambskin from Hu sheep is world famous for its wavy pattern and is known as a “soft gem” [1]. The production of Hu sheep lambskin has increased due to its increased market demand. However, in recent years, attention has been focused on meat characteristics rather than the quality of lambskin during the selection process, resulting in a gradual decrease in lambskin quality over time, which in turn has caused significant economic losses. Therefore, identifying, developing, and protecting unique germplasm resources to provide high-quality genetic material for breeding is of great economic value.

The quality of lambskin is affected by various factors such as types, visibility, and distribution area of pattern. These indices are generally used to evaluate the quality of lambskin. Fineness, density, and curvature of the hair follicles, in turn, determine the type of wave pattern [2]. Therefore, wool quality is based on pattern formation, and hair follicle characteristics form the basis for hair growth [3].

The hair follicle is a complex accessory organ of the skin that has a unique morphology and structure. It controls skin growth and plays a major function in skin regeneration. Hair follicles consist of epithelial and hypodermal layers, which include the connective tissue sheath, inner root sheath, outer root sheath, hair bulb, and hair shaft [4]. Hair follicles are divided into primary follicles and secondary follicles. The primary follicles form coarse wool, and secondary follicles produce the fine wool known as fluff [5]. The relative density of primary and secondary follicles determines the fineness and curvature of wool, thus affecting the quality of wool. Hair follicles in most mammals has a periodic regularity of development, including a growth stage, a regression stage, and a telogen phase [6,7]. Various types of cell participate in the biological processes for the regulation of this cyclical development [8,9], which is influenced by several regulatory pathways and molecules. Signaling pathways such as Wnt [10], TGF-β [11,12], MAPK [13], Shh [14], and Notch [15] are extensively involved in different components of hair follicle morphogenesis and its periodic variation. In addition, some cytokines also play an important role in hair follicle development and hair growth processes, including KGF [16], IGF [17], VEGF [18], EGF [19], and thymosin β4 [20], which promote the growth of hair follicles, and FGF-5 [21,22], which inhibits hair growth. MicroRNAs (miRNAs) are endogenously expressed, highly conserved small RNAs with 21–25 nucleotides that are derived from one arm of their precursor with a stem-loop structure that is expressed in specific cells and tissues [23]. Landgraf et al. [24] reported that miRNAs are differentially expressed in abnormal physiology and developmental processes. Viswanathan et al. [25] observed that miRNA can regulate RNA-induced silencing complex (RISC) by mRNA shearing and inhibition. It regulates the expression of target genes that participate in physiological and pathological processes by interacting with the target mRNA’s 3’-untranslated region. In recent years, studies have shown that miRNAs are also expressed in hair follicles of mammals. Yi et al. [26] constructed a small RNA library from the skin and hair follicles of E17.5 fetal rats and reported that it predominantly consisted of miRNAs. In addition, by knocking out the key gene *Dicer* I that controls miRNA maturation, they discovered that mice fail to develop hair follicles. Mardaryev et al. [27] discovered that 200 miRNAs were expressed differentially in different growth stages of hair follicles. Yuan et al. [28] identified 399 known miRNAs and 172 new miRNAs in Cashmere goat skin. Around 326 miRNAs were expressed at different stages of hair follicle development and 26, 41, and 55 miRNAs were expressed during the growth stage, regression stage, and telogen phase, respectively. Liu et al. [29] identified differentially expressed miRNAs at 3 development stages of Tibetan sheep and found that miRNAs regulated the growth of hair follicles by the regulation of target genes in the MAPK and Wnt pathway.

There is currently very little information on miRNA expression during hair follicle development, and no relevant miRNA study on hair follicles has been conducted; therefore, the molecular mechanisms underlying the formation of hair follicles with different wave patterns are not currently known. Transcriptome sequencing was used to study the molecular mechanism underlying the formation of these types of hair follicles in Hu sheep and to screen differentially expressed miRNAs and genes that were related to the process of proliferation and differentiation, growth, and apoptosis of hair follicle cells. The present study may establish a theoretical basis for the molecular mechanism underlying the formation of different wave patterns in the hair follicles of Hu sheep.

## Results

### Overview of miRNA and mRNA sequencing results

We sequenced the miRNA and mRNA from the total RNA samples including large-wave wool, medium-wave wool, and small-wave wool using an Illumina Hiseq 2500 sequencer. From the miRNA libraries, the number of clean reads in large-wave wool, medium-wave wool, and small-wave wool were 69,657,452, 6,609,614, and 6,422,619, respectively. From the mRNA libraries, the number of clean reads in large-wave wool, medium-wave wool, and small-wave wool were 67,172,566, 71,812,980, and 69,133,870, respectively. Detailed results of sequencing and assembly are shown in Tables 1 and 2.

**Table 1:** Summary of miRNA sequencing.

Sample name	Raw reads	Clean reads	Aligned reads	Unaligned reads
Large	7964087	6965742	4897877	1605824
Medium	7167843	6609614	4970514	1639100
Small	7534406	6422619	5359918	1524742

**Table 2:** Summary of mRNA sequencing.

Sample name	Raw Reads	Raw Bases(bp)	Valid Reads	Valid bases(bp)	Valid base
WGC020580_Large	70364532	7036453200	67172566	6674922377	94.86%
WGC020580_Medium	72238464	7223846400	69133870	6870414871	95.10%
WGC020580_Small	75029244	7502924400	71812980	7135638732	95.10%

We obtained 522 differentially expressed miRNAs by analyzing the expression level of each miRNA in the gene library using DESeq, and then significant analysis of microarrays was used to select the significantly and differentially expressed miRNAs in the 3 types of pattern. The results showed that there were 24 significantly and differentially expressed miRNAs in small and medium waves, and compared to small waves, medium waves contained 18 downregulated miRNAs and 5 upregulated miRNAs. There were 18 significantly and differentially expressed miRNA in medium waves and large waves, including 3 downregulated miRNAs and 14 upregulated miRNAs compared to medium waves. There were 13 significantly and differentially expressed miRNAs in small waves and large waves, including 7 downregulated miRNAs and 4 upregulated miRNAs compared to small waves. These results showed that there were 36 novel miRNAs and 6 known miRNAs that were differentially expressed in lambskin (Table 3).3333A total of 751, 745, and 764 differentially expressed genes were detected between small and large waves, medium and large waves, and small and medium waves, respectively. Analysis of differentially expressed genes between any two patterns indicated that there were 481 downregulated genes and 270 upregulated genes in large waves compared to that observed in small waves, 380 downregulated genes and 365 upregulated genes in medium waves compared to that observed in small waves, 493 downregulated genes and 271 upregulated genes in large waves compared to that in medium waves. (S1 Table). In addition, we determined that MAPK8 (XM_004021553.1) and ROCK2 (XM_004007209.1) were associated with the Wnt signaling pathway. These findings indicate that differentially expressed genes might be involved in hair follicle growth and development.

**Table 3:** Significantly differentially expressed miRNAs in large, medium, and small waves.

miR-name	Small read count	Medium read count	Large read count	Log2 (FC)	P-value	Regulated	Sig
NW_004080165.1_8884	10	1		-3.6866	0.0307	Down	*
NW_004080164.1_4714	10	1		-3.6866	0.0307	Down	*
NW_004080166.1_9842	30	4		-3.2715	0.0096	Down	**
NW_004080164.1_5170	121	19		-3.0356	0.0053	Down	**
oar-miR-10a	539	99		-2.8094	0.0013	Down	**
NW_004080184.1_6535	150	33		-2.5491	0.0128	Down	*
NW_004080174.1_726	508	112		-2.5460	0.0034	Down	**
NW_004080183.1_5976	27	6		-2.5346	0.0357	Down	*
NW_004080165.1_8572	6,207	1,405		-2.5080	0.0002	Down	**
NW_004080172.1_13048	64	15		-2.4578	0.0301	Down	*
NW_004080184.1_6326	7,666	1,953		-2.3374	0.0003	Down	**
NW_004080164.1_5130	104	27		-2.3102	0.0306	Down	*
NW_004080172.1_12940	297	83		-2.2039	0.0152	Down	*
NW_004080165.1_8918	319	96		-2.0970	0.0197	Down	*
NW_004080166.1_10417	255	110		-2.0550	0.0199	Down	*
NW_004080165.1_8917	317	101		-2.0148	0.0241	Down	*
NW_004080181.1_3961	2,478	799		-1.9976	0.0066	Down	**
NW_004080166.1_9139	4,299	1,399		-1.9842	0.0041	Down	**
NW_004080182.1_4070	30	153		1.9859	0.0458	Up	*
NW_004080177.1_2323	29	148		1.9868	0.0469	Up	*
NW_004080177.1_2324	27	139		1.9994	0.0478	Up	*
NW_004080176.1_1908	6	46		2.5739	0.0280	Up	*
NW_004080186.1_7163	1	14		3.4427	0.0380	Up	*
oar-miR-200a	6	0		Int	0.0332		*
NW_004080190.1_13734		2,414	1,157	-1.1893	0.0265	Down	*
NW_004080190.1_13733		2,415	1,160	-1.1862	0.0268	Down	*
oar-let-7i		8,030	4,286	-1.0340	0.0392	Down	*
NW_004080166.1_9139		1,399	4,871	1.6715	0.0410	Up	*
NW_004080184.1_6326		1,953	8,010	1.9078	0.0158	Up	*
NW_004080172.1_12940		83	341	1.9103	0.0477	Up	*
NW_004080165.1_8918		96	417	1.9907	0.0321	Up	*
NW_004080181.1_3961		799	3,508	2.0061	0.0127	Up	*
NW_004080165.1_8572		1,405	7,641	2.3149	0.0028	Up	**
NW_004080166.1_10417		110	602	2.3240	0.0083	Up	**
NW_004080174.1_726		112	632	2.3681	0.0069	Up	**
NW_004080164.1_5708		17	110	2.5656	0.0383	Up	*
NW_004080170.1_12254		9	63	2.6791	0.0422	Up	*
NW_004080168.1_11242		15	109	2.7330	0.0281	Up	*
NW_004080166.1_9842		4	43	3.2980	0.0171	Up	*
NW_004080167.1_10826_star		1	15	3.7786	0.0387	Up	*
NW_004080795.1_13255		6	135	4.3636	0.0007	Up	**
NW_004080190.1_14262		0	35	Inf	0.0005		**
oar-miR-199a-3p	19		3	-3.16	0.0028	Down	**
NW_004080177.1_2600	11		3	-2.37	0.0322	Down	*
NW_004080184.1_6535	150		70	-1.5925	0.0115	Down	*
NW_004080189.1_7961	273		142	-1.4359	0.0089	Down	**
oar-let-7c	243		150	-1.1889	0.0264	Down	*
oar-miR-10a	539		335	-1.1790	0.0141	Down	*
oar-miR-143	5,967		3,835	-1.1307	0.0046	Down	**
NW_004080166.1_9447	53		259	1.7960	0.0328	Up	*
NW_004080166.1_10400	21		133	2.1700	0.0259	Up	*
NW_004080165.1_8152	7		63	2.6770	0.0163	Up	*
NW_004080795.1_13255	2		135	5.5839	6.03E-06	Up	**
NW_004080181.1_3973	6		0	Int	0.0159		*
NW_004080190.1_14262	0		35	Inf	0.0003		**

Note: The threshold of significantly expressed miRNAs is Log_2_(FC) > 1 and *p* < 0.01; down: in small waves and medium waves, compared to small waves, the miRNA in medium waves were downregulated miRNAs; in medium waves and large waves, compared to medium waves, the miRNAs in large waves were downregulated miRNAs; in small waves and large waves, compared to small waves, the miRNAs in large waves were downregulated miRNAs; up: the miRNAs were upregulated

*: *p* < 0.05

**: *p* < 0.01

FC: fold change.

### Cluster analysis of differentially expressed miRNAs

MiRNA with similar expression patterns usually have similar biological functions, and cluster analysis is generally used to analyze the expression levels of differentially expressed miRNAs in different patterns. The differentially expressed miRNAs from three types of patterns were analyzed by hierarchical cluster analysis (S1–S3 Figs). Expression pattern of miRNA were classified into the same cluster in 3 types of patterns, but the expression quantity of miRNAs between any 2 patterns had major differences; therefore, we speculated that there were various regulatory mechanisms that were involved in different patterns.

### MiRNA target prediction and integration of miRNA and mRNA expression profiles

We ran the miRanda [30] and TargetScan [31] prediction software to study the biological functions of 42 differentially expressed miRNAs. The results showed that there were 43, 81, and 190 target genes in small and large waves, medium and large waves, and small and medium waves, respectively. We further integrated the sequencing data into the predicted miRNA-mRNA pairs to validate the target pairs. A total of 20, 52, 45 target pairs were found to be inversely expressed.

### Functional annotation and classification

Gene ontology (GO) and biological pathway enrichment were done for the 130 genes [32,33]. Gene ontology analysis showed that 4, 25, and 7 GO items were significantly enriched with these genes in small and large waves, medium and large waves, and small and medium waves, respectively (Tables 4, 5 and 6). These GO items were enriched with biological processes, cellular component and molecular function. In addition, these genes were mainly involved in cell differentiation, proliferation, apoptosis, growth, immune response, and ion transport.

**Table 4:** Gene ontology analysis based on miRNA-targeted differentially expressed genes in small waves and large waves.

GO id	GO term	GO category	p-value
GO:0031013	Troponin I binding	Function	0
GO:0045095	keratin filament	Component	2.22E-05
GO:0005198	structural molecule activity	Component	4.46E-03
GO:0005882	intermediate filament	Component	3.92E-03

**Table 5:** Gene ontology analysis based on miRNA-targeted differentially expressed genes in small waves and medium waves.

GO id	GO term	GO category	p-value
GO:0042998	positive regulation of Golgi to plasma membrane protein transport	Process	0
GO:0071253	connexin binding	Function	0
GO:0005802	trans-Golgi network	Component	6.89E-3
GO:0005886	plasma membrane	Component	1.43E-2
GO:0043234	protein complex	Component	2.16E-2
GO:0030133	transport vesicle	Component	2.98E-2
GO:0010923	negative regulation of phosphatase activity	Process	3.61E-2

**Table 6:** Gene ontology analysis based on miRNA-targeted differentially expressed genes in medium waves and large waves.

GO id	GO term	GO category	p-value
GO:1901029	negative regulation of mitochondrial outer membrane permeabilization	Process	0
GO:0043408	regulation of MAPK cascade	Process	3.47E-4
GO:0006665	sphingolipid metabolic process	Process	1.11E-3
GO:0019216	regulation of lipid metabolic process	Process	1.11E-3
GO:0060742	epithelial cell differentiation involved in prostate gland development	Process	1.11E-3
GO:0071310	cellular response to organic substance	Process	1.11E-3
GO:0015280	ligand-gated sodium channel activity	Function	2.56E-3
GO:0050896	response to stimulus	Process	2.56E-3
GO:0060736	prostate gland growth	Process	2.56E-3
GO:0034706	sodium channel complex	Component	6.30E-3
GO:0050699	WW domain binding	Function	6.30E-3
GO:0005272	sodium channel activity	Function	6.30E-3
GO:0006810	transport	Process	6.30E-3
GO:0006814	sodium ion transport	Process	7.74E-3
GO:0005764	lysosome	Component	7.74E-3
GO:0005764	lysosome	Component	7.74E-3
GO:0050909	sensory perception of taste	Process	8.48E-3
GO:0006811	ion transport	Process	1.08E-2
GO:0035725	sodium ion transmembrane transport	Process	1.08E-2
GO:0005794	Golgi apparatus	Component	1.08E-2
GO:0005794	Golgi apparatus	Component	1.08E-2
GO:0048589	developmental growth	Process	1.65E-2
GO:0043231	intracellular membrane-bounded organelle	Component	3.04E-2
GO:0043231	intracellular membrane-bounded organelle	Component	3.04E-2
GO:0005765	lysosomal membrane	Component	3.99E-2

Pathway analysis identified 5, 16, and 12 signaling pathways in small and large waves, medium and large waves and small and medium waves, respectively (Tables 7, 8 and 9). In addition, the mTOR signaling pathway and the MAPK signaling pathway were connected with hair follicle development. At the same time, many cancer pathways were overrepresented within these genes, such as the FoxO signaling pathway. Some genes were also enriched in pathways that were related to hair follicle growth and development such as the Wnt signaling pathway, the Notch signaling pathway, and the TGF-beta signaling pathway. Although the enrichment in these 3 pathways was not significant, these genes remain important topics for future investigations.

**Table 7:** Pathway analysis based on miRNA-targeted differentially expressed genes in small waves and large waves.

KEGG id	KEGG description	p-value
ko04141	Protein processing in endoplasmic reticulum	4.13E-3
ko05203	Viral carcinogenesis	1.32E-2
ko05218	Melanoma	1.32E-2
ko00590	Arachidonic acid metabolism	2.57E-2
ko04150	mTOR signaling pathway	4.92E-2

**Table 8:** Pathway analysis based on miRNA-targeted differentially expressed genes in small waves and medium waves.

KEGG id	KEGG description	p-value
ko00062	Fatty acid elongation	1.83E-2
ko00720	Carbon fixation pathways in prokaryotes	1.83E-2
ko00660	C5-Branched dibasic acid metabolism	1.83E-2
ko01210	2-Oxocarboxylic acid metabolism	2.57E-2
ko01230	Biosynthesis of amino acids	4.01E-2
ko05110	Vibrio cholerae infection	4.01E-2
ko04966	Collecting duct acid secretion	4.09E-2
ko04145	Phagosome	4.10E-2
ko00620	Pyruvate metabolism	4.24E-2
ko00290	Valine	4.24E-2
ko00970	Aminoacyl-tRNA biosynthesis	4.24E-2
ko05323	Rheumatoid arthritis	4.57E-2

**Table 9:** Pathway analysis based on miRNA-targeted differentially expressed genes in medium waves and large waves.

KEGG id	KEGG description	p-value
ko05205	Proteoglycans in cancer	1.43E-2
ko05120	Epithelial cell signaling in Helicobacter pylori infection	1.43E-2
ko04150	mTOR signaling pathway	1.43E-2
ko05203	Viral carcinogenesis	1.43E-2
ko05110	Vibrio cholerae infection	1.43E-2
ko04966	Collecting duct acid secretion	1.43E-2
ko05219	Bladder cancer	1.87E-2
ko04068	FoxO signaling pathway	1.87E-2
ko04722	Neurotrophin signaling pathway	1.96E-2
ko05223	Non-small cell lung cancer	2.13E-2
ko03060	Protein export	3.07E-2
ko04720	Long-term potentiation	3.23E-2
ko04919	Thyroid hormone signaling pathway	3.42E-2
ko04010	MAPK signaling pathway	3.46E-2
ko04721	Synaptic vesicle cycle	3.98E-2
ko05214	Glioma	4.80E-2

### Differentially expressed miRNAs and mRNA identified by RT-PCR analysis

To confirm the small RNA and mRNA sequencing results, 5 miRNAs and mRNA were selected for RT-PCR verification. Although the results of sequencing and PR-PCR analyses indicated different fold changes, the expression trends were the same. This finding indicates that the sequencing results were reliable (Tables 10 and 11).

**Table 10:** Comparison of the results of sequencing and RT-PCR analyses of differentially expressed miRNAs.

Small RNA sequencing			RT-PCR	
miRNA	Group	Log2(FC)	Log2(FC)	*P* value
miRNA-143	1	-0.131	-1.120	0.022*
	2	-0.032	-0.305	0.690
	3	-0.802	-0.816	0.038*
let-7c	1	-1.189	-1.181	0.041*
	2	-0.687	-0.720	0.426
	3	-0.491	-0.461	0.014*
NW_004080189.1_7961	1	-1.436	-1.142	0.047*
	2	-0.778	-0.347	0.855
	3	-0.658	-0.800	0.061
NW_004080181.1_3961	1	0.008	0.011	0.001**
	2	-1.995	-2.251	0.002**
	3	2.006	2.260	<0.001**
NW_004080166.1_10417	1	0.270	0.310	0.002**
	2	-2.055	-1.841	0.020*
	3	2.232	2.148	<0.001**

Note: 1, small and large waves; 2, small and medium waves; 3, medium and large waves

**Table 11:** The comparisons on the results of sequencing and RT-PCR analyses of differentially expressed genes.

mRNA sequencing			RT-PCR	
gene	Group	Log2(FC)	Log2(FC)	*P* value
LMO4	1	-4.132	-4.237	<0.001**
	2	-0.428	-0.477	0.908
	3	-3.724	-3.775	<0.001**
IGF2BP2	1	0.601	0.263	0.341
	2	-0.269	-0.288	0.188
	3	0.851	0.549	0.045*
FGF1	1	-2.912	-2.943	<0.001**
	2	-0.418	-0.479	0.925
	3	-2.514	-2.457	<0.001**
MAPK8	1	-0.517	-0.720	0.015*
	2	-0.905	-1.486	0.048*
	3	0.367	0.764	0.407
MT4	1	0.181	0.124	0.193
	2	0.775	0.841	0.008**
	3	-0.614	-0.717	0.002**

### Regulatory network analysis of miRNA-mRNA

**Fig 1:**
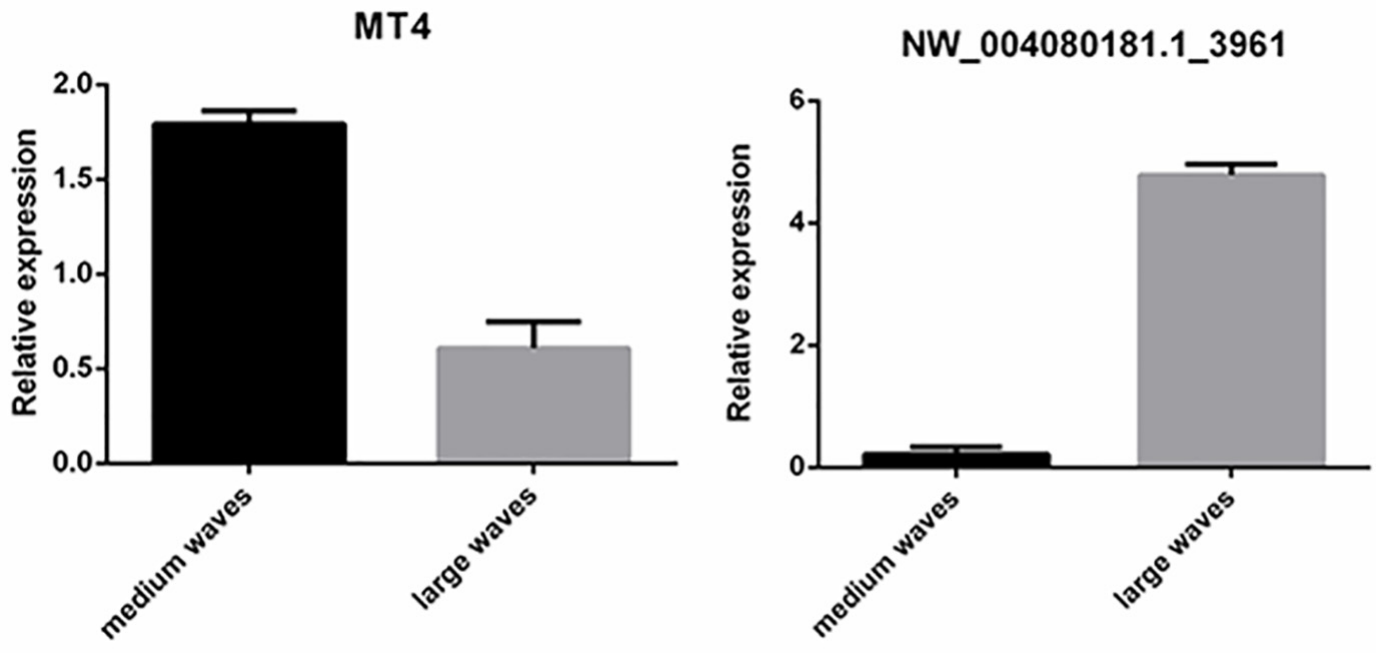
RT-PCR expression on miRNA and gene.

In organisms, miRNAs play a role in post-transcriptional regulation by suppressing or silencing specific target genes. Therefore, we looked for correlations between upregulated miRNAs and downregulated mRNAs, as well as between downregulated miRNAs and upregulated mRNAs. The results demonstrate the changes in the number of differentially expressed miRNAs from small RNA sequencing and differentially expressed genes from transcriptome sequencing. Compared to small waves, there were 7 differentially expressed miRNAs, including 4 downregulated miRNAs and 3 upregulated miRNAs, and 20 differentially expressed genes, including 12 downregulated genes and 8 upregulated genes in large waves (S2 Table). Compared to small waves, there were 19 differentially expressed miRNAs, including 15 downregulated miRNAs and 4 upregulated miRNAs, and 52 differentially expressed genes, including 7 downregulated genes and 45 upregulated genes in medium waves (S3 Table). Compared to medium waves, there were 12 upregulated miRNAs and 41 downregulated genes. (S4 Table). Among these miRNAs and genes, NW_004080181.1_3961 and MT4 were selected for RT-PCR validation of miRNA and mRNA co-expression (Fig 1). The results showed that 1 upregulated miRNA, NW_004080166.1_10417, was predicted to target the downregulated MAPK8. Additionally, the downregulated miRNA, NW_004080181.1_3961, was predicted to target the upregulated MT4. In addition, NW_004080164.1_5130 and IGF2BP2, oar-miR-10a and TCEB1, oar-miR-200a and PABPC4, etc. may play roles in hair follicle development.

## Discussion

Hu sheep lambskin is one of the most well-known in the world and is prized for several specific attributes, including its wave pattern, white color, soft hair, clarity, and beauty. It contains 3 kinds of waves, namely large, medium, and small, which are based on the peak distances in the wave patterns. The quality of small waves is considered far superior to that of large waves. In the present study, we used high-throughput sequencing to screen miRNAs and genes that were differentially expressed in large-wave, medium-wave, and small-wave follicles. To adjust for differences between individuals and environmental factors, we evaluated full-sibling sheep. Gene ontology was used to divide genes into 3 categories, namely cellular component, molecular function, and biological progress. The differentially expressed genes observed in the present study were mainly involved in development, cell differentiation, proliferation, apoptosis, immune response, and ion transport. Furthermore, pathway analysis indicated that specific genes were involved in the Wnt, TGF-β, and MAPK signaling pathways. These highly expressed genes may also be involved in hair growth and hair follicle development.

### Predictive analysis of miRNAs and target genes

Studies have shown that miRNA accounts for 2%-5% of mammalian genes, and it regulates 60% of protein-coding genes [34]; therefore, miRNAs are the most widely distributed regulatory factor in animals [35]. Previous studies have found that miRNAs regulate protein expression through translation inhibition, which in turn influences various developmental processes, including muscle development, hair follicle development, and mammary gland development [36]. In the body, miRNAs exert their biological functions by targeting specific genes. In the present study, we determined that miRNAs regulate multiple target genes, whereas a target gene may be regulated by multiple miRNAs. The relationship between miRNAs and target genes plays an important role in the regulation of hair follicle growth and pattern formation, thus establishing it as a very good method for studying miRNAs [37]. In the present study, we identified 522 differentially expressed miRNAs and through significance analysis, 36 unknown miRNAs and 6 known miRNAs were identified. Among these miRNAs, miR-10 was determined to regulate the transcription of the Hox gene, as well as various activities involved in protein synthesis, whereas miR-10 possibly plays an important role in cell development and cell differentiation [38]. MiR-let-7, which includes miR-let-7i, miR-let-7c, and miR-let-7b, is expressed in the skin of goats, sheep, and mice, and its target genes were related to the growth of hair follicles and hair quality. Members of the MiR-200 family were expressed in the epidermis and hair follicles, whereas those of the miR-199 family were only expressed in hair follicles. MiR-199a-3p, which was a member of the miR-199 family, reduces cell proliferation in cancer and suppressed the occurrence of cancer [39], thus, we speculated that miR-200a and miR-199a-3p possibly play a role in hair follicle growth and development. In addition, Trakooljul et al. found that most target genes of miR-143 were related to cell proliferation, apoptosis, and tumorigenesis [40].

### The association analysis of differentially expressed miRNAs and genes

MiRNAs have a negative regulatory relationship with target genes, and can inhibit and degrade the expression of target genes. We have analyzed the expression of miRNAs and genes between pairs of patterns in Hu sheep, including a combination of both differentially expressed miRNA and genes, and we have examined the regulatory network of differentially expressed miRNA and genes to identify additional target genes.

To study the effect of differentially expressed genes on different wave patterns in the hair of Hu sheep, association analysis was adopted to examine the expression and regulatory mechanism of differentially expressed miRNAs and genes during pattern formation, including upregulated miRNAs and downregulated genes as well as downregulated miRNAs and upregulated genes. Most miRNA-regulated target genes and differentially expressed genes are novel miRNAs that regulate various genes such as those expressed in small waves and large waves. For example, NW_004080189.1_7961 has a regulatory relationship with differentially expressed genes such as *NFATC3*, *KPNB1*, *NRN1*, and *MEF2A*. Gene ontology analysis showed that these genes were mainly involved in cell differentiation, proliferation, apoptosis, growth, immune response, and ion transport. *LMO4*, the target gene of miR-143, regulates EMT that is caused by *TGF-β*. EMT participates in various physiological and pathological processes such as the regeneration of damaged tissue, tissue fibrosis, tumorigenesis, and tumor metastasis [41]. FGF is involved in the process of cell migration and cell development, and it is a molecule that significantly promotes angiogenesis. *FGF1*, which is the target gene of NW_004080189.1_7961, is a member of the fibroblast growth factor (FGF) family, thus, it could also significantly promote angiogenesis and cell division, as well as participate in the growth and development of various tissues and organs. *FGF1* plays a very important role in cell proliferation, migration, and differentiation [42,43,44]. *IGF2BP2*, the target gene of miR-let-7i, regulates the translation of *IGF-2* [45]. *IGF-2* is a member of the insulin-like growth factor system (IGFs), and *IGF-2* stimulates cell proliferation and affects the growth and differentiation of tissues and organs to promote tumor formation. *TCF4* is the target gene of NW_004080182.1_4070 and NW_004080177.1_2324. *TCF4* is often combined with β-catenin of the Wnt signaling pathway and is recognized as the most important molecule of the T-cell factor [46]. *TCF4* restrains the Wnt signaling pathway because some isomers of *TCF4* lack the binding site for β-catenin [47], and yet some studies have shown that *TCF4* activates the Wnt pathway, and may be related to the source of the cells or tissues [48]. Some studies have shown that *MT3* is the encoding gene of metallothionein in cells; it has a unique molecular structure and high affinity for metal ions, and not only has the physiological function of other MTs, but also inhibits cell physiological activities, as well as acting as a suppressor gene [49]. Although it also belongs to the MT family, *MT4* was determined not to be involved in the inhibition of cell proliferation. It could be also used as an important candidate gene because it is differentially expressed in hair follicles. KEGG pathway analysis showed that target genes were enriched in the cancer pathway, as well as other pathways related to hair follicle development such as the Wnt, TGF-β, MAPK, and mTOR signaling pathways. Su et al. [50] reported that miR-200a directly or indirectly affects the expression of β-catenin, as well as participates in the Wnt signaling pathway. In addition, miR-199a-3p influences the activity of the mTOR signaling pathway. Differentially expressed genes *MAPK3* and *MAPK8* are involved in the MAPK signaling pathway, and these have specific regulatory functions. In addition, *TCF4* is regulated by internal signals in the Wnt pathway.

## Materials and Methods

### Ethics statement

The Institutional Animal Care and Use Committee (IACUC) of the government of Jiangsu Province (Permit Number: 45) and the Ministry of Agriculture of China (Permit Number: 39) approved the animal study proposal. All experimental procedures were conducted in strict compliance with the recommendations of the Guide for the Care and Use of Laboratory Animals of Jiangsu Province and of the Animal Care and Use Committee of the Chinese Ministry of Agriculture. All efforts were made to minimize animal suffering.

### Experimental populations

Three pairs of full-sib individuals were selected at birth from among Hu sheep reared at a Suzhou stud farm in China and the farm owners gave permission for use of these sheep. Each pair contained 1 individual with predominantly large-wave, 1 individual medium-wave wool, and 1 with predominantly small-wave wool. About 1 cm of hair root was cut off and placed in a microtube surrounded by Drikold, and the quantity of hair root was collected 1/3 of the volume of the microtube, which was then collected into the freezing tube and stored in Drikold.

### Total RNA extraction and cDNA library preparation

Total RNA was isolated using the Recover All Total Nucleic Acid Isolation Kit (Life Technologies, Carlsbad, CA, USA) for miRNA and mRNA sequencing, according to the manufacturer’s instructions. Integrity of RNA was checked on an Agilent 2100 bioanalyzer. The sequencing library was prepared according to the standard protocol. Briefly, for miRNA sequencing, libraries were prepared by ligating different adaptors to the total RNA followed by reverse transcription and PCR amplification (RT-PCR). MiRNA libraries were sequenced on the Illumina Hiseq2500 system with 50-base-pair (bp) single-end reads, according to the manufacturer’s standard protocol. All small sequencing raw data were deposited in the Sequence Read Archive database (SRR3099013, SRR3099014, and SRR3099015). For mRNA sequencing, total RNA was firstly poly-A-selected followed by fragmentation of RNA into small pieces. The cleaved RNA fragments were reverse transcribed to cDNA, end-repaired, and ligated with Illumina adapters using a Quick ligation TM kit (NEB) and DNA ligase. The libraries were then fractionated on agarose gel; short fragments (approximately 200 bp) were excised and amplified by PCR. The cDNA library was sequenced on the Illumina sequencing platform, and raw reads were generated using the Solexa pipeline according to the manufacturer’s recommendations. All mRNA sequencing raw data were deposited in the Sequence Read Archive database (SRR3099016, SRR3099017, and SRR3099018).

### Reads processing

For small sequencing reads, all low quality reads such as those with poly A/T and adapter contaminants were filtered. Sequences longer than 40 bp or shorter than 15 bp were removed. The high-quality clean reads were mapped to the *Ovis aries* genome using Bowtie software. Small RNA tags were aligned to the miRNA precursor/mature miRNA in miRBase. Furthermore, Rfam, RepBase and Genbank data were used to identify small RNA tags originating from miRNA, rRNA, tRNA, snRNA, and snoRNA.

For mRNA sequencing reads, adaptor sequences and low quality reads (threshold quality, 20; threshold length, 35 bp) were filtered. The matched reads were aligned to *Ovis aries*. The mRNA expression level was measured by FPKM (fragments per kilobase per million reads).

### Real-time PCR verification

Differentially expressed miRNAs and genes were randomly selected, and U6 and GAPDH were used as reference genes. The SYBR Green I method was used for quantitative testing to verify the reliability of the sequencing results. Relevant information about genes that were assessed via RT-PCR analysis and the primers used in the assay are shown in S5 Table. The standard curve was established using a cDNA gradient dilution, and each sample was tested 3 times in a 7500 PCR instrument. The relative expression of the target gene was calculated according to the following formula [51]: Relative expression = 2^-ΔΔ^Ct, ΔΔC t = (C, target gene—C, housekeeping gene)_large waves_−(C t, target gene—C, housekeeping gene)_small waves_. SPSS17.0 was used to compute for the Ct mean and standard deviation between replicate samples, and one-way ANOVA was adopted for analysis of significance.

## Supporting Information

S1 Fig Hierarchical clustering of differentially expressed miRNAs between small waves and large waves. (TIF)

S2 Fig Hierarchical clustering of differentially expressed miRNAs between small waves and medium waves. (TIF)

S3 Fig Hierarchical clustering of differentially expressed miRNAs between medium waves and large waves. (TIF)

S1 Table Significantly differentially expressed mRNAs in large, medium, and small waves. (XLSX)

S2 Table Correlation analysis of miRNA and mRNA between small waves and large waves. (XLSX)

S3 Table Correlation analysis of miRNA and mRNA between small waves and medium waves. (XLSX)

S4 Table Correlation analysis of miRNA and mRNA between medium waves and large waves. (XLSX)

S5 Table Primers used in real-time RT-PCR analysis. (XLSX)
