# Supplementary figures and images for: An Integrated Analysis of MicroRNA and mRNA Expression Profiles to Identify RNA Expression Signatures in Lambskin Hair Follicles in Hu Sheep
Source: PLoS One. 2016 Jul 12;11(7):e0157463. doi: 10.1371/journal.pone.0157463 (PMC4942090; doi:10.1371/journal.pone.0157463)

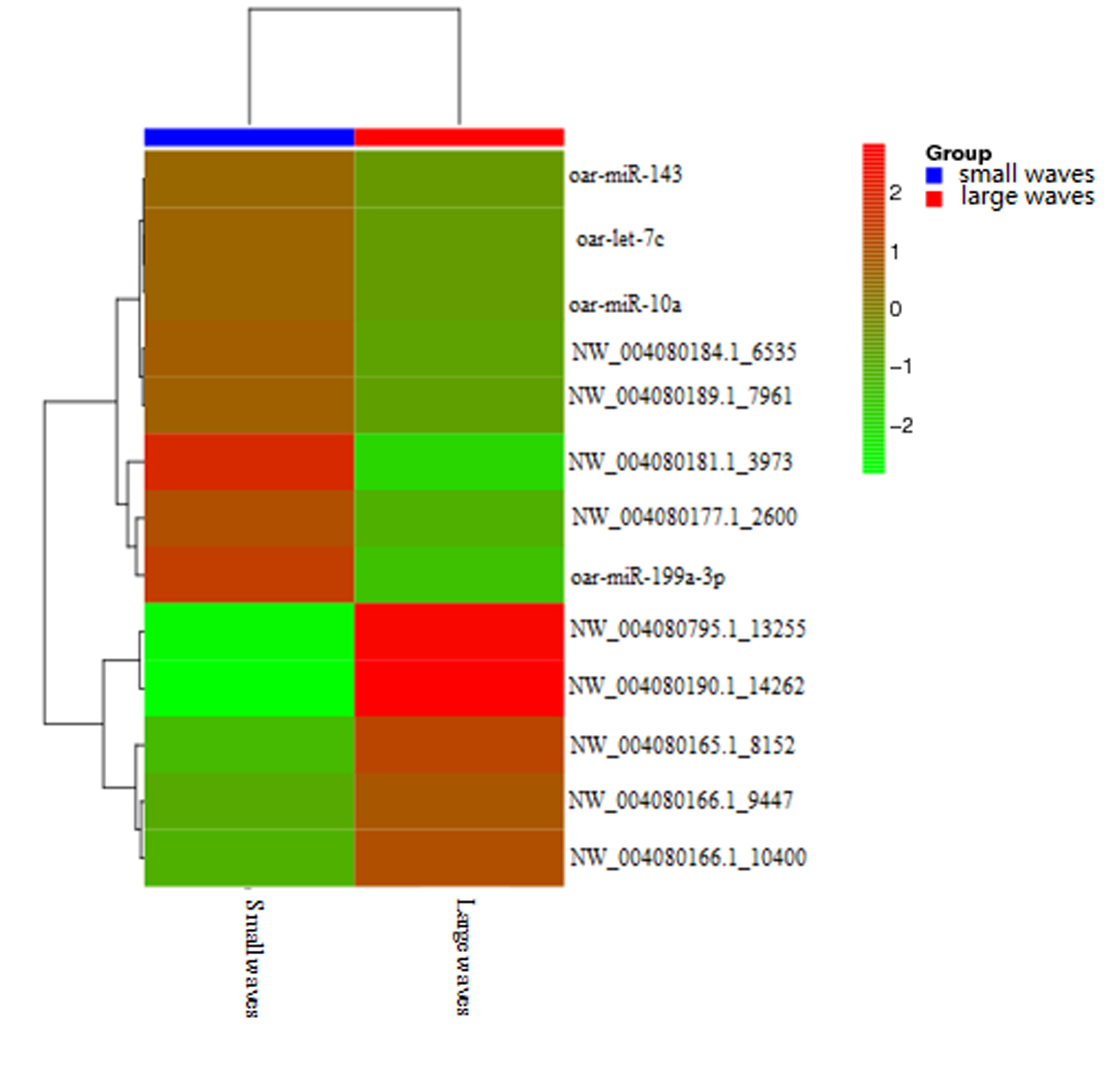

Supplement: S1 Fig — (TIF) [file pone.0157463.s001.tif]

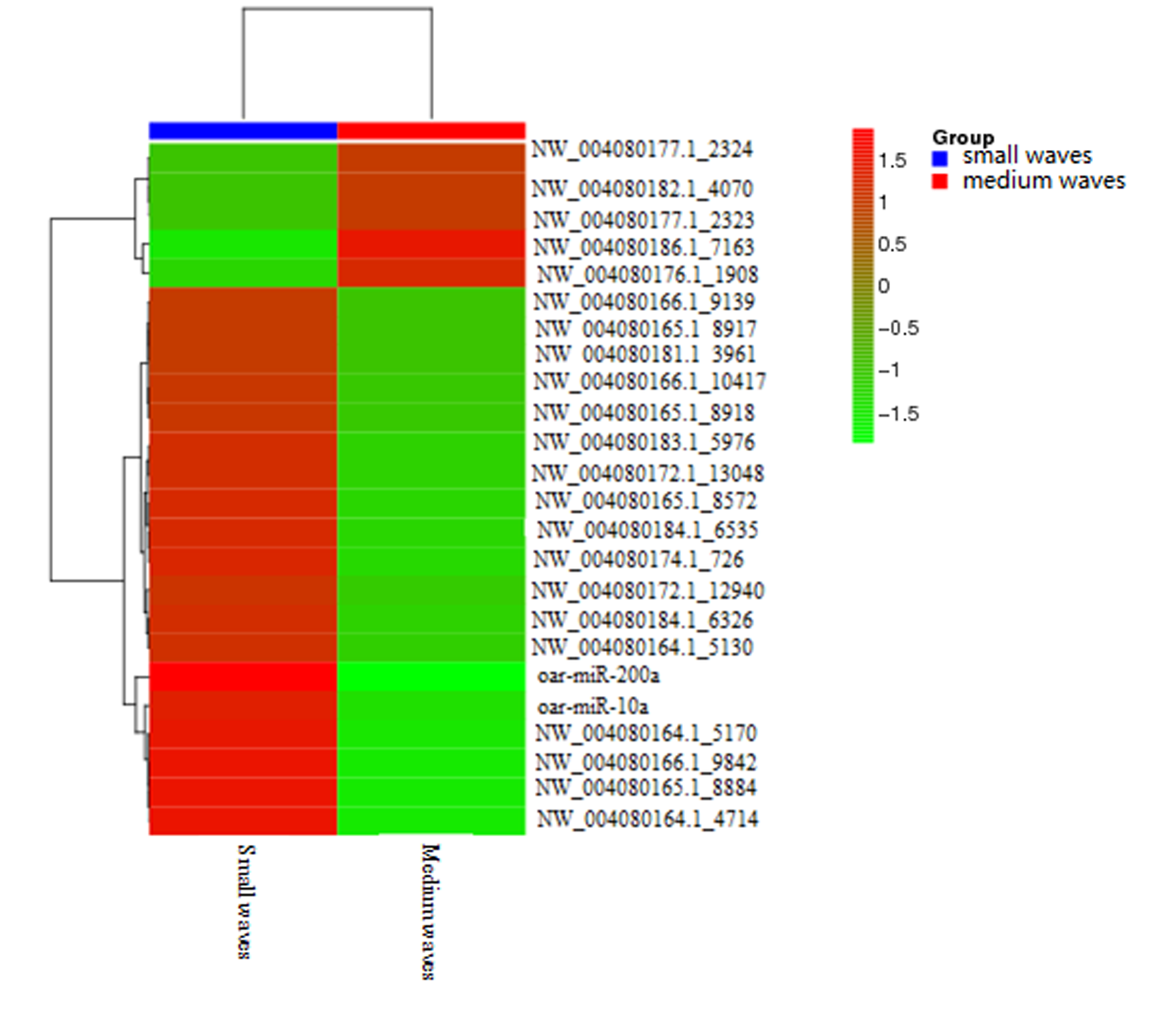

Supplement: S2 Fig — (TIF) [file pone.0157463.s002.tif]

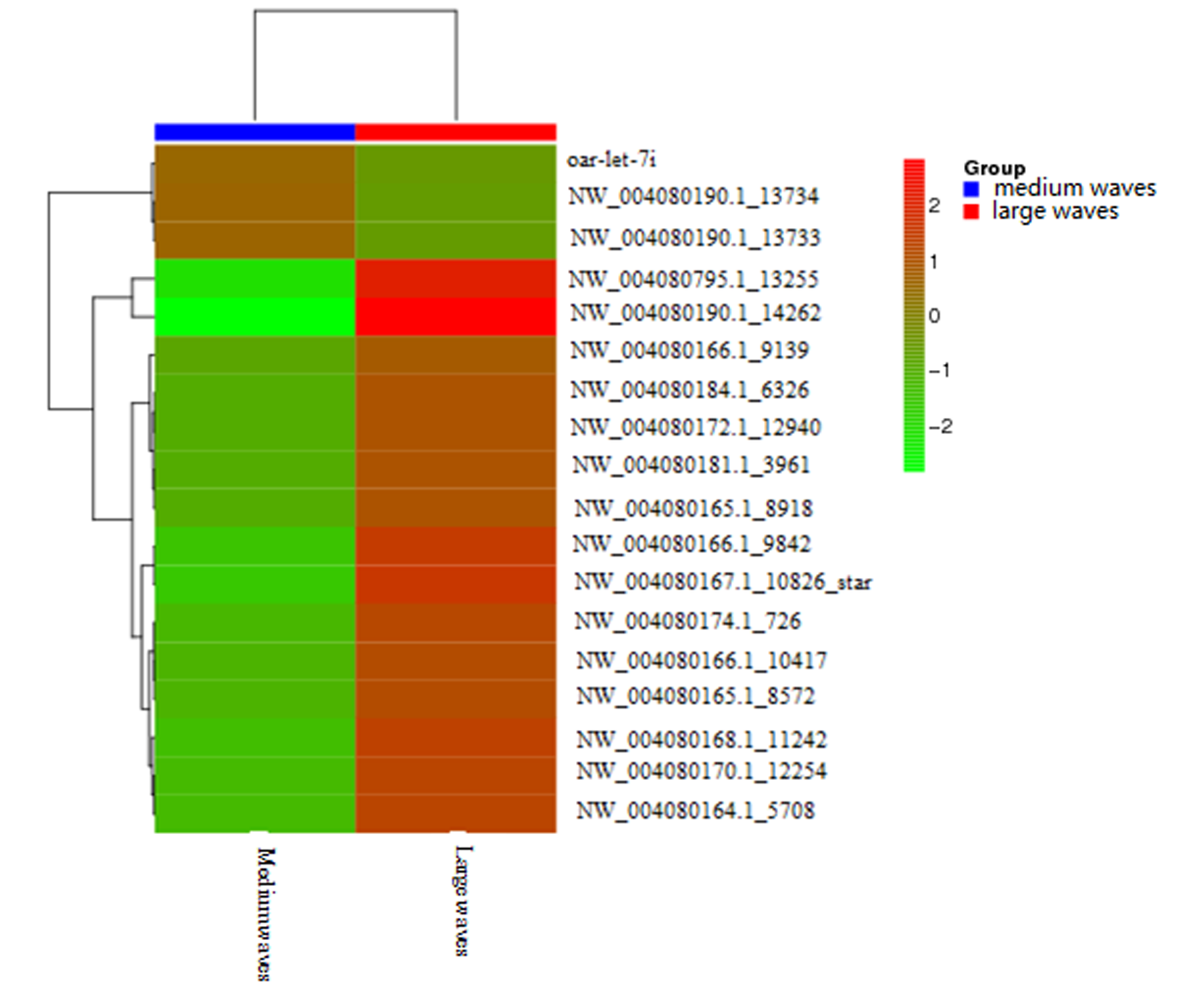

Supplement: S3 Fig — (TIF) [file pone.0157463.s003.tif]
